# Impact of Climatic Factors on the Seasonal Fluctuation of Leishmaniasis Vectors in Central Morocco (Meknes Prefecture)

**DOI:** 10.1155/2020/6098149

**Published:** 2020-12-24

**Authors:** Hajar El Omari, Abdelkader Chahlaoui, Fatima Zahra Talbi, Karima EL Mouhdi, Abdelhakim El Ouali Lalami

**Affiliations:** ^1^Natural Resources Management and Development Team, Laboratory of Health and Environment, Faculty of Sciences, Moulay Ismail University, Meknes, Morocco; ^2^Laboratory of Biochemistry, Neurosciences, Natural Resources and the Environment, Faculty of Sciences and Technologies, Hassan First University, BP 577, Settat, Morocco; ^3^Geo-biodiversity and Natural Heritage Laboratory, GEOPAC Center, Scientific Institute-Rabat, University Mohamed V Morocco, Rabat, Morocco; ^4^Institute of Nursing Professions and Health Techniques of Fez, Regional Health Directorate, EL Ghassani Hospital, Fez 30000, Morocco

## Abstract

The impact of climate factors on the epidemiology of diseases in general and leishmaniasis in particular continues to be a subject of research and analysis. Changes in climatic parameters contribute to the creation of ecological conditions favorable to the multiplication of the vectors of certain diseases. With this in mind, this study presents an entomological survey conducted in Meknes prefecture and the study of the link between the abundance of sandflies, an indicator of the risk of leishmaniasis in a given area, and the climatic factors. Monthly trapping of this fauna was carried out during a year from March 2016 until April 2017 using adhesive traps. Climatic data from the region were used to determine the effect of climate on the distribution of sandflies. A total of 941 leishmaniasis vector specimens were captured. The dominant species is *Ph. sergenti* (73.32), followed by *Ph. longicuspis* (8.25%), then *Ph. perniciosus* (7.94%) and *Ph. papatasi* (6.31%). The sex ratio study showed that males are more abundant than females for all species. The seasonal fluctuation is bimodal with two peaks, the first in July and the second in September. The results show a positive correlation between temperature and abundance of sandflies (*r* = 0.99) and a negative correlation with humidity and precipitation with a correlation coefficient of *r* = −0.87 and *r* = −0.72. Indeed, the medium-term climatological forecasts are essential tools to develop a warning system for leishmaniasis.

## 1. Introduction

Sandflies are the only vectors of leishmaniasis that are common diseases between animals and humans and caused by parasites Leishmania protozoa [1, 2]. The impact of climatic factors on the spread of vector-borne diseases including leishmaniasis is a particular concern. The incidence of infectious diseases like leishmaniasis will tend to increase in warmer climatic conditions.

The expansion of the most warmed regions will be accompanied by the geographical expansion of these diseases [3, 4]. Generally, the vectors of this epidemic live in warmer regions, especially in tropical and subtropical regions between 50°N and 40°S4. Many sandfly species are established in Europe [5–8], and their main distribution area can be found in the Mediterranean region, e.g., Portugal, Italy, Spain, Croatia, and Greece [9]. Indeed, the climatic factors influence the distribution of this disease [10, 11].

As in most countries around the Mediterranean, leishmaniasis in Morocco constitutes a serious public health problem [12] because of its clinical and epidemiological diversity [13]. Leishmaniasis in these different forms are endemic in many areas, each with its own vectors and reservoirs [14, 15]; the number of cases reported in 2015 by the Epidemiology Department of the Moroccan Ministry of Public Health is 2 900 cases of leishmaniasis.

In order to determine the effect of climate on the dynamics of the vectors of leishmaniasis, a study of the seasonal fluctuation of the phlebotomian population and its relationship with climatic factors was carried out from April 2016 to March 2017, in the prefecture of Meknes in the center of Morocco. Vector abundance could be used as an indicator of the risk of leishmaniasis in a given area [16].

## 2. Materials and Methods

### 2.1. Study Area

**Figure 1:**
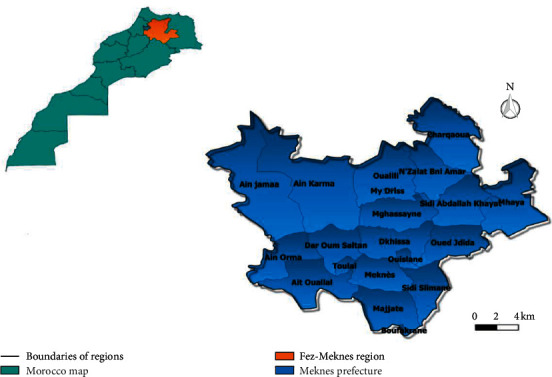
Geographical location of the study area.

The prefecture of Meknes (Figure 1) is located northwest of Morocco in the Meknes-Fes region, about 130 km from the capital of Morocco and 60 km from the city of Fes. The altitude of the city is about 500 m.

It occupies a strategic geographical position because, on the one hand, it is located between two sets of mountains: Pre Rif and the Western Middle Atlas and, on the other hand, thanks to the positioning of the city of Meknes at the crossroads of major communication routes between the different cities of the Kingdom of Morocco.

In the prefecture of Meknes, two types of leishmaniasis coexist: visceral and cutaneous leishmaniasis that might have propagated from Taounate to the foci of Meknes and Sidi-Kacem via Fez, with stray dogs carrying the infection across hamlets and villages in their quest for food and shelter [17].

### 2.2. Data Acquisition

In our study, we performed two sandflies capture sessions monthly from April 2016 to March 2017 using castor oil impregnated papers (adhesive traps) in six different biotopes chosen according to the proximity to the agglomerations and the epidemiological history of localities [18]. The study stations represent sites or biotopes favorable to the development of sandflies (egg-laying and/or resting environment). They are essentially stables and sheepfolds, but also human dwellings and caves.

The traps were set in the evening, and they were recovered the next morning to prevent deterioration of the sandflies.

The captured insects were identified based on the key to determine sandflies in Morocco. The meteorological data of the year of study were obtained from the meteorological station of the Agropolis station of Meknes.

### 2.3. Data Processing

For the statistical processing of data, we used the correlation coefficient which gives information about the existence of a linear relationship (in the form of a straight line) between two quantities considered. We calculated the correlation index by the following formula:

(1) $$\begin{array}{c} r=\frac{\sum \left(X-\bar{X}\right).\left(Y-\bar{Y}\right)}{\sqrt{{\sum \left(X-\bar{X}\right)}^{2}}\times \sqrt{\sum {\left(Y-\bar{Y}\right)}^{2}}}. \end{array}$$

The correlation coefficient is between −1 and +1. The further away from zero, the correlation is better.

To study the impact of climate factors on different species of sandflies during the months of catches, we performed principal component analysis (PCA), using SPSS software (7.0).

## 3. Results

### 3.1. Entomological Survey

In our study, 941 sandfly specimen species divided into three subgenus were harvested. The entomological analyses show that the recovered species are *Ph. sergenti* (76.51), *Ph. longicuspis* (8.61%), *Ph. perniciosus* (8.29%), and *Ph. papatasi* (6.59%) (Table 1).

For the sex ratio, we note that, for all the species, there was a dominance of the males compared to the females with a sex ratio of 3.44.

### 3.2. Dynamics of Sandflies

**Figure 2:**
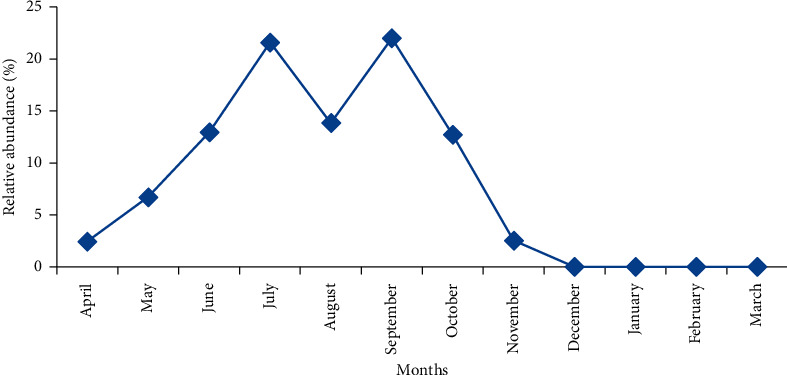
Seasonal fluctuation of sandflies.

**Figure 3:**
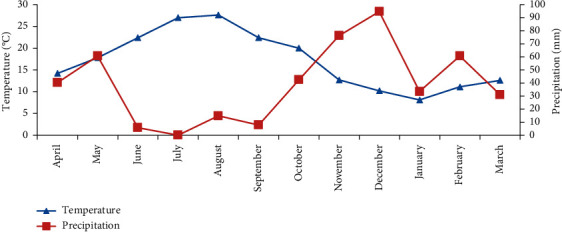
Ombrothermic diagram of the study area.

The seasonal fluctuation of phlebotomian fauna follows a bimodal curve (Figure 2). It shows that the activity period lasts eight months from April to November with two peaks, the first in July and the second in September. This period coincides with the dry period of the year characterized by the increase of temperature and the decrease of precipitations (Figure 3).

### 3.3. Impact of Climatic Factors

**Figure 4:**
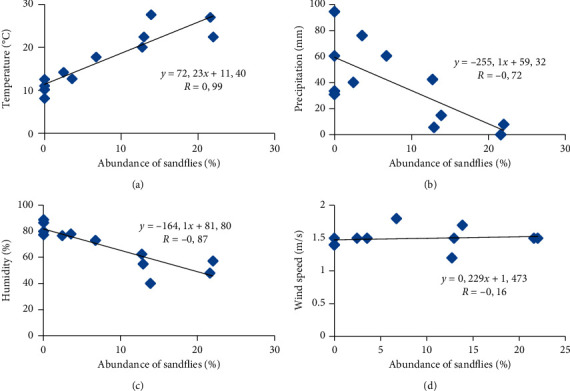
Correlation between the abundance of sandflies and temperature (a), precipitation (b), humidity (c), and wind speed (d).

The study of the impact of climatic factors on the abundance of this fauna shows the existence of a highly positive correlation between temperature and the different phlebotomian species (*r* = 0.99) (Figure 4(a)). However, this correlation is strongly negative between the precipitation and the abundance of this fauna (*r* = −0.72) (Figure 4(b)).

We also note from Figures 4(c) and 4(d) that the correlation is strongly negative between humidity and phlebotomine activity with a correlation index *r* = −0.87, whereas the correlation between the wind speed and abundance of sandflies is negative and low (*r* = −0.16).

**Figure 5:**
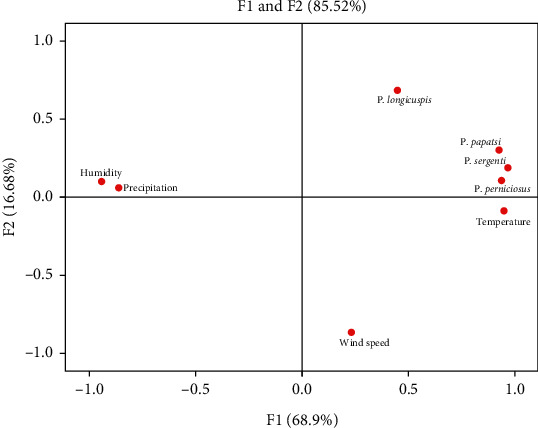
Result of principal component analysis (PCA).

Similar results have been proved by principal component analysis (PCA). Indeed, the analysis of results by principal component (PCA) shows that the first two components can be considered for the explanation of the variability of the data because they explain most of the information (Figure 5).

The two axes taken into account to describe the correlation between the variables related to the spatial structures represent 85.52% of the total information, with, respectively, 68.9% for F1 and 16.62% for F2.

The analysis of the factorial planes F1 and F2 shows that the values of the temperature and the different species of sandflies are positively related both with each other and with the axis F1, whereas the precipitation and humidity are linked together and are negatively correlated on the F1 axis.

## 4. Discussion

The present study is an entomological study of the phlebotomian population and the effect of climate on the distribution of these insects.

A total of 4 species were captured, *Ph. papatasi* proved as *L. major* vector [19–21], *Ph. sergenti* the only vector of *L. tropica*, *Ph. perniciosus*, a proven vector of canine and human visceral leishmaniasis [22, 23], and *Ph. longicuspis* strongly suspected as a vector of *L. infantum* [24, 25]. The last entomological works carried out in Morocco confirm the presence of the species found in our studies [26–28].

Constituting 76.83% of the total workforce, males are predominant compared to females, which represent only 23.17% of the population. This difference between the number of males and females is partly due to the capture means used (adhesive traps) and the type of biotope prospected. These results are corroborated with other research, which has confirmed the dominance of the males by contribution to the female [29, 30].

The seasonal fluctuation of phlebotomian fauna follows a bimodal curve. It shows that the activity period lasts eight months from April to November with two peaks, the first in July and the second in September. A recent study in the Mediterranean region has confirmed that peaks increasing in magnitude were frequently observed from July through September [31].

Indeed, the fluctuation and abundance of phlebotomine species are related to various factors including climatic factors [32]. Their periods of activity and abundances are largely conditioned by climatic factors, particularly temperature and precipitation. They are distributed according to their bioclimatic affinities [33].

The significant period of activity of sandflies in our study is between June and October. This period coincides with the dry period of the year characterized by increasing temperature and decreasing precipitation.

The statistical analysis shows that the correlation is highly significant between rainfall and the abundance of sandflies and that these two factors are correlated between them and also with the F1 axis.

The temperature favors the multiplication of these insects while increasing their activities and their frequencies, which reinforces the vectorial capacity of the sandflies and the transmission of leishmaniasis. However, the precipitation inhibits its proliferation.

The results of our study are consistent with the findings of other researchers around the world [34, 35], who confirmed that increasing temperature increases vector proliferation and also promotes vectorial capacity, thus facilitating transmission of vector diseases.

Similar results have been proved in Senegal, by the work of Desjeux et al. [36], who show that variations in climatic factors cause variations in the abundance of sandflies, and according to this study, phlebotomian activity begins at the time when the temperature increases, However, the rains cause the decrease of the density which confirms the results of our study.

In addition, the study carried out in Mediterranean countries confirms that temperature whose magnitude is negatively correlated with latitude is a major determinant for the activity start of leishmaniasis vectors. It is well established that, during cold months, sand flies undergo diapause as fourth larval stage (L4) [31].

It should also be noted that an increase in temperature increases the rate at which pathogenic elements mature and multiply in the animal [37, 38], which increases the chances of transmission of parasitic diseases, like leishmaniasis, especially as vector insects proliferate faster and sting more in warmer air.

Indeed, with the warm season tending to lengthen, unlike the cold season, which tends to shorten [39], sandflies vectors of leishmaniasis may become more abundant and extend their range. Long, hot summers are also ideal conditions for Diptera [40]; thereafter, the risk of leishmaniasis increases.

It would appear as well that the correlation is negative between the precipitation, the humidity, and the activity of the sandflies (*r*). Consequently, these two factors inhibit the activity of the sandflies; these results are consistent with the data found in Spain [41], which confirms that the sufficiently high humidity of the air accelerates the death of the larvae and subsequently the disappearance of adults. On the other hand, results in Colombia show the positive impact of precipitation on sandflies [42].

The factorial analysis shows that the temperature values and the different species of sandflies are positively related both to each other and to the F1 axis while precipitation and humidity are interrelated as well as negatively correlated with the F1 axis.

Therefore, temperature and precipitation are two limiting factors that determine the importance of vector activity and subsequently the occurrence and evolution of leishmaniasis. However, this relation remains relative since one can find exceptions which can be explained by the intervention of the other factors which can be related to pathogenic complex (parasite, vector, and reservoir) and the presence of the favorable environment for the multiplication of the vectors.

For the wind speed, it is found that it is on the other axis (F2), so it has no significant effect on the distribution of these insects; in addition, the correlation between the speed of the wind and the different species of sandflies remained low and this could be explained by the small variation in wind speed during the year of search.

## 5. Conclusion

The present study allowed us to determine the different species vectors of leishmaniasis and also the periods of activity of sandflies in the prefecture of Meknes.

The high abundance of *Ph. sergenti*, a vector of leishmaniasis of *L tropica*, in the study area, presents a high risk in this province.

Our study also reveals that changes in temperature, precipitation, and humidity would affect the biology and ecology of vectors, therefore the risk of disease transmission. The study of the correlation index as well as the circle of correlation shows that the period of activity of sandflies is highly determined by the climatic conditions of the environment, and we have shown that the density of this fauna is quite important when the temperatures are maximum and rainfall is minimum (dry period of the year), and during the rainy months, no species was captured. The different species of sandflies can live in temperate conditions, whereas they cannot survive in the wet and rainy season.

## Figures and Tables

**Table 1:** Presence and relative abundance of species in Meknes prefecture.

Genus	Subgenus	Species	Male	Female	Total	Relative abundance (%)
*Phlebotomus*	*Larroussius*	*Ph. perniciosus*	61	17	78	8.29
		*Ph. longicuspis*	68	13	81	8.61
	*Paraphlebotomus*	*Ph. sergenti*	560	173	720	76.51
	*Phlebotomus*	*Ph. papatasi*	50	12	62	6.59
	Total		739	215	941	100

## Data Availability

The data used to support the findings of this study are included within the article.
